# Current Hospital Topics

**Published:** 1906-10-27

**Authors:** 


### HOSPITAL ADMINISTRATION.

### CONSTRUCTION AND ECONOMICS.

### CURRENT HOSPITAL TOPICS.

#### The Royal Free Hospital.

The Royal Free Hospital has long and deservedly possessed the confidence and the sympathy of most intelligent givers to medical charities. It was the first hospital to admit all patients free and without tickets, and in recent years the economy and excellence of its administration have commended it to the warm support of men of business who take the trouble to make themselves familiar with hospital facts. The Royal Free Hospital has a system of investigation which carefully sifts the patients who apply for relief, and takes infinite pains to prevent any possibility of abuse in the out-patient department. Following out this policy a little further the Board has just determined to examine into the circumstances of every in-patient admitted to treatment for a definite period, and all will look forward to the results of this investigation with genuine interest. The Royal Free Hospital is unfortunately at the moment sadly in need of money, and H.R.H. the Duke of Connaught has consented to preside at the festival dinner in aid of its funds to be held at the Hôtel Cecil on November 28, 1906. We hope, despite the bad times, that this dinner will prove a record success; for there is no hospital in London which has stronger claims upon the liberality and support of the more intelligent and generous contributors to hospitals.

#### The Value of Balconies in Hospitals.

The reconstruction of the larger general hospitals in great cities in this country has led to the addition of large balconies where patients may be treated when in bed. This addition has greatly improved the conditions of treatment for many cases, and has had a marked tendency in expediting recovery and reducing the number of days residence of such patients. At the Cardiff Infirmary Viscount Tredegar has defrayed the cost of erecting verandahs some eight feet wide and fifty feet long, which face west. These verandahs are connected with every important ward, and have been in use for two years. The results are so satisfactory that the medical board have moved the managers to inform Viscount Tredegar of the great benefit his generous gift has conferred on the patients in the Cardiff Infirmary. The use of these verandahs, the medical board state, has rendered immense service in promoting and accelerating the recovery of many patients medically and surgically treated in the open air, besides conducing greatly to the comfort of the patients and the happiness of many others. Such undoubted testimony to the practical benefits to be derived from the addition of verandahs to modern hospitals should lead many wealthy people in inquire whether the hospital in their immediate neighbourhood has verandahs. If it has not, they could not find a better use for their money than to offer to defray the cost of providing these modern aids to treatment with as little delay as possible.

#### Funds and Voluntary Hospitals.

There is a general complaint that it is becoming more and more difficult to raise money for voluntary hospitals in London. Appeals through the Press have been overdone, and the response is frequently found to be so small as to barely pay for the outlay entailed by this method of raising money. Then the festival dinner on the old lines fails to attract, and is found in fact to be increasingly laborious and unsatisfactory as the years pass. We pointed out a new, and, as has been proved, a better method of raising funds in The Hospital of March 3, 1906. The truth is that the continuous absence of business in the City of London for years past has produced its effect, and compelled the average giver to considerably reduce his expenditure including his gifts to charities. Then too there is an increasing feeling amongst the more wealthy and intelligent supporters of hospitals that free medical relief is overdone, and that it is incumbent upon the management of every large hospital to reduce its volume in accordance with the demands of the general practitioners, with whom the public have much sympathy. The existence of this feeling and its effect upon contributions to hospitals may be ascertained by anyone who will take the trouble to analyse the later contributions from the more wealthy congregations to the Hospital Sunday Fund, and to compare them with those in previous years. They will find such an examination will show that the contributions from the wealthier congregations, where there are very few poor requiring hospital tickets, is decreasing, although the contributions from the poorer congregations show an upward tendency. This fact justifies the effort which Lord Kilmorey is systematically making in support of Charing Cross Hospital. The principle which underlies it is that every large hospital in a big city should first of all attend to the requirements of the poor in the district in which it is situated, and then claim regular support for the hospital from all classes resident in that district. We believe Lord Kilmorey is likely to meet with a surprising success in his present effort, and if this be the case there is little doubt that other hospitals which require money will follow a similar system. At the same time it must be borne in mind that the actual amount of money given to the support of the London hospitals from voluntary sources at the present time is, collectively, infinitely greater than it has ever been before in the history of these institutions. Men of business who have gone carefully into the matter are convinced that the voluntary hospitals in London, as a whole, have at present sufficient funds to meet the cost of granting medical attendance to every man, woman and child who can properly claim hospital aid when ill. The raising of funds therefore assumes a twofold aspect, and its successful accomplishment entails a change of policy in two directions also. First of all the management must secure, with the aid of the general practitioners, the hearty co-operation of the visiting medical staffs to reduce materially the amount of free medical relief at present given; secondly arrangements must be made to provide that the patients who apply from the district in which the hospital is situated first receive the in- or out- patient relief to which they are entitled, and then, but then only, can consideration be given to strangers and outsiders. Once secure this point, and an organisation on Lord Kilmorey's plan should speedily put every London hospital in sufficient funds to do its work with the utmost efficiency.

